# Entropy Generation Analysis of Hybrid Nanomaterial through Porous Space with Variable Characteristics

**DOI:** 10.3390/e23010089

**Published:** 2021-01-10

**Authors:** Muhammad Adil Sadiq, Farwa Haider, Tasawar Hayat

**Affiliations:** 1 Department of Mathematics, DCC-KFUPM, Dhahran 31261, Saudi Arabia; 2 Department of Mathematics Quaid-I-Azam University, Islamabad 45320, Pakistan; farwahaider3@gmail.com (F.H.); fmgpak@gmail.com (T.H.)

**Keywords:** hybrid nanofluid (MoS_2_ and SiO_2_), Darcy–Forchheimer–Brinkman porous space, non-linear thermal radiation, viscous dissipation, heat generation/absorption, ND Solve

## Abstract

Salient features of hybrid nanofluid (MoS_2_-SiO_2_/water) for Darcy–Forchheimer–Brinkman porous space with variable characteristics is examined. Heat transfer analysis subject to viscous dissipation, nonlinear thermal radiation, and heat generation/absorption is carried out. Disturbance inflow is created by an exponentially stretching curved sheet. Relevant equations are simplified by employing boundary layer theory. Adequate transformations lead to a set of dimensionless equations. Velocity, temperature, and entropy generation rate are analyzed graphically. Comparative results are obtained for hybrid (MoS_2_-SiO_2_/water) and nanofluid (MoS_2_-water and SiO_2_-water). Physical quantities are analyzed through numerical data.

## 1. Introduction

Electronics, automotive, telecommunication, aerospace, and biomedical industries require microdevices for heat transfer enhancement in a system. Heat transfer efficiency of such devices can be improved by using a working fluid with enhanced thermophysical properties like thermal conductivity and specific heat. Hybrid nanofluids are potential materials produced by dispersing two dissimilar nanoparticles (metals, carbide and oxide ceramics, carbon nanotubes, and metals) in base fluid (ethylene glycol, oil, and water). Such fluids have superior thermophysical properties and thermal performance than nanofluids. Such nanofluids save energy as well as less harmful environmental impacts. After the pioneering work of Choi [1] on nanofluids, several studies have been conducted to analyze the behavior of such materials. Few of these are mentioned here which considered different nanoparticles such as Cu, Al_2_O_3_, Ag, CuO, and several others. Eastman et al. [2] analyzed improvement in thermal conductivity of ethylene glycol-based copper nanofluid. It is noted that ethylene-glycol based copper nanofluid has much higher effective thermal conductivity than pure ethylene glycol. The flow of nanofluid in a lid-driven square cavity is provided by Tiwari and Das [3] They analyzed the behavior of nanofluid by considering solid volume fraction of nanoparticles. Vajravelu et al. [4] presented convective heat transfer in Ag-water and Cu-water nanofluids. A comparative analysis is performed for Ag-water and Cu-water nanofluids. It is observed that boundary layer thickness decreases more rapidly in the case of Ag-water nanofluid in comparison to Cu-water. The three-dimensional flow of nanofluid is examined by Khan et al. [5]. Devasenan and Kalaiselvam [6] provided an experimental investigation of the heat transfer behavior of hybrid nanofluid. Copper-titanium hybrid nanocomposites are considered. They found an increase in thermal conductivity due to the highly crystalline nature of copper-titanium hybrid nanofluid. Malvandi et al. [7] discussed mixed convection in Al_2_O_3_-water nanofluid. Selimefendigil et al. [8] elaborated mixed convection in SiO_2_-water nanofluid by a rotating cylinder. Different shapes of nanoparticles are considered such as spherical, cylindrical, brick, and blade. It is analyzed that the heat transfer rate of cylindrically shaped nanoparticles is higher than that of others. Improvement in heat transfer of Ag-CuO/water nanofluid is addressed by Hayat and Nadeem [9]. Iqbal et al. [10] analyzed curvilinear transport of MoS_2_-SiO_2_/water nanofluid. It is noted that blade-shaped nanoparticles have maximum temperature while brick-shaped nanoparticles have the lowest temperature. Thermally radiative flow of Cu-Al_2_O_3_/water nanomaterial over a permeable surface is interpreted by Usman et al. [11] Mansour et al. [12] provided entropy generation analysis of square porous cavity filled with Al_2_O_3_-Cu/water nanofluid. The influence of internal heat generation in the flow of MoS_2_-SiO_2_/C_3_H_8_O_2_ is studied by Shaiq et al. [13] Khan et al. [14] analyzed entropy generation analysis of MoS_2_-SiO_2_/C_3_H_8_O_2_ nanofluid with variable viscosity. Heat transfer enhancement in hybrid nanofluid along the wavy surface is studied by Iqbal et al. [15]. It is noted that hybrid nanofluid has a higher transfer rate than nanomaterial. Khan et al. [16] presented an entropy generation analysis of MoS_2_-SiO_2_/ water nanofluid through porous space. Acharya [17] analyzed the behavior of hybrid nanofluid inside a microchannel. Hydromagnetic flow of Cu-Al_2_O_3_/water nanofluid past moving sheet is illustrated by Aladdin et al. [18] Flow of hybrid nanofluid saturating porous medium with mixed convection is discussed by Waini et al. [19] Aly and Pop [20] provided comparative analysis for stagnation point flow of hybrid nanofluid and nanomaterial with MHD. 

Porous space is composed of interconnected solid particles and pores generally encountered in electrochemical systems, iron and steel making, microchemical reactors, biofiltration systems, and combustion of carbon-neutral and renewable fuels. Extensive theoretical and computational studies about porous media are based on classical Darcy’s law. To include inertia and viscous diffusion effects in Darcy’s law, the modifications are made by Forchheimer [21] and Brinkman [22,23] respectively. To resolve this paradox, Nield [24] modeled viscous dissipation in a porous medium. Hadhrami et al. [25] provided another model for viscous dissipation in porous space. Mixed convective flow through porous space is analyzed by Seddeek [26] Umavathi et al. [27] illustrated Darcy-Forchheimer-Brinkman flow of nanofluid in a vertical rectangular duct. Latest developments in flow through a porous medium with constant porosity and permeability and can be cited through refs. [28,29,30,31,32,33,34,35,36]. However little information is available for variable characteristics of porous space [37,38,39,40,41,42,43,44]

Entropy generation is a quantitative tool based on the second law of thermodynamics. It measures irreversibilities in the fluid flow process. Heat and mass transfer, viscous dissipation, buoyancy, and magnetic field are the source of chaos in a thermal system. Several studies are conducted to anticipate the entropy generation rate in thermal systems followed by the pioneering work of Bejan [45]. Entropy generation of nanofluid in a cavity is analyzed by Mahmoudi et al. [46]. It is observed that the entropy generation rate decreases due to the addition of nanoparticles. Entropy generation analysis of nanofluid in a vertical porous microchannel is provided by López et al. [47] Sithole et al. [48] explored entropy generation analysis of nanofluid with nonlinear thermal radiation. It is noted that the entropy generation rate decreases in presence of thermal radiation. Entropy generation analysis of ferrofluid saturating porous space is elaborated by Astanina et al. [49]. Huminic and Huminic [50] discussed entropy generation analysis of hybrid nanofluid. Entropy generation analysis of viscous fluid with buoyancy is interpreted by Ganesh et al. [51] Kashyap and Dass [52] deliberated entropy generation analysis of the two-phase mixed convective flow of hybrid nanofluid. The effects of three different boundary conditions on fluid flow are analyzed. It is observed that the entropy generation rate increases by a change in the boundary condition. Moreover, the addition of nanoparticles also augments the entropy generation rate which is not desirable for the effectiveness of a thermal system. Entropy generation for nanofluid through non-Darcy porous space is studied by Sheikholeslami et al. [53] Effect of activation energy inflow over the curved surface with entropy generation is analyzed by Muhammad et al. [54] Hayat et al. [55] provided entropy generation for the flow of nanofluid due to curved surface filling porous space. Hayat et al. [56] presented entropy generation analysis of effective Prandtl number.

In view of the above-mentioned studies, the main objectives of the present study are threefold. Firstly, to formulate the flow of hybrid nanofluid by a curved stretching surface through porous space. Variable porosity and permeability are chosen. This concept is given a little attention even for flow by flat stretching case. Secondly, to consider the effects of nonlinear thermal radiation and heat generation/absorption in heat transfer analysis. Thirdly to anticipate the entropy generation rate in the considered problem. Solution development is due to the NDSolve technique of Mathematica. Characteristics of flow, thermal field, and entropy generation rate through involved variables are interpreted. Numerical computations are obtained for physical quantities.

## 2. Model Development 

**Figure 1:**
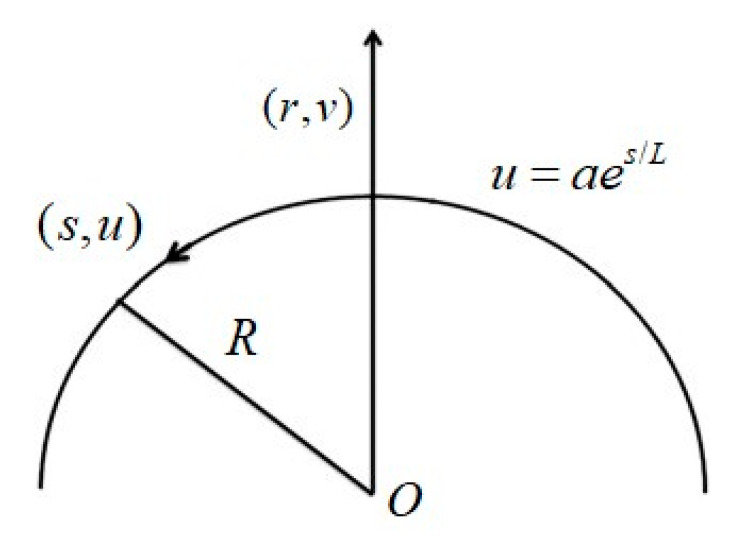
Physical model of flow configuration.

Here the flow of hybrid nanofluid through Darcy–Forchheimer–Brinkman porous space is analyzed. Viscous dissipation, heat generation/absorption, and non-linear thermal radiation are also taken. The disturbance in flow is created by a curved stretching surface. The sheet is stretched with an exponential velocity $u_{w}\left(s\right)=ae^{s/L}$ (see Figure 1). Here curvilinear coordinates frame (s, r) is adopted. Relevant equations for the considered problem are

(1) $$\frac{\partial }{\partial r}\left(\left(r+R\right)v\right)+R\frac{\partial u}{\partial s}=0,$$

(2) $$\frac{u^{2}}{r+R}=\frac{1}{\rho_{hnf}}\frac{\partial p}{\partial r},$$

(3) $$\begin{array}{c} v\frac{\partial u}{\partial r}+\frac{R}{r+R}u\frac{\partial u}{\partial s}+\frac{uv}{r+R}=-\frac{1}{\rho_{hnf}}\frac{R}{r+R}\frac{\partial p}{\partial s}+\\ \nu_{hnf}\left(\frac{\partial^{2}u}{\partial r^{2}}+\frac{1}{r+R}\frac{\partial u}{\partial r}-\frac{u}{{\left(r+R\right)}^{2}}\right)-\nu_{hnf}\frac{\varepsilon \left(r\right)}{k^{*}\left(r\right)}u-\frac{C_{b}\varepsilon^{2}\left(r\right)}{{\left(k^{*}\left(r\right)\right)}^{1/2}}u^{2}, \end{array}$$

(4) $$\begin{array}{cc} v\frac{\partial T}{\partial r}+u\frac{\partial T}{\partial s}\frac{R}{r+R}= & \alpha_{hnf}\left(\frac{\partial^{2}T}{\partial r^{2}}+\frac{1}{r+R}\frac{\partial T}{\partial r}\right)+\frac{\mu_{hnf}}{{\left(\rho c_{p}\right)}_{hnf}}{\left(\frac{\partial u}{\partial r}-\frac{u}{r+R}\right)}^{2}+\\ \; & \frac{Q}{{\left(\rho c_{p}\right)}_{hnf}}\left(T-T_{\infty }\right)-\frac{1}{{\left(\rho c_{p}\right)}_{hnf}}\frac{\partial }{\partial r}\left(-\frac{16\tilde{\sigma }}{3k}T^{3}\frac{\partial T}{\partial r}\right)+\frac{\mu_{hnf}}{{\left(\rho c_{p}\right)}_{hnf}}\frac{\varepsilon \left(r\right)}{k^{*}\left(r\right)}u^{2}+\\ \; & \frac{\rho_{hnf}}{{\left(\rho c_{p}\right)}_{hnf}}\frac{C_{b}\varepsilon^{2}\left(r\right)}{{\left(k^{*}\left(r\right)\right)}^{1/2}}u^{3}, \end{array}$$

(5) $$u=ae^{s/L},\;v=0,\;T=T_{f}=T_{\infty }+T_{0}e^{As/2L}\;\mathrm{at}\;r=0,$$

(6) $$u\to 0,\;\frac{\partial u}{\partial r}\to 0,\;T\to T_{\infty }\;\mathrm{as}\;r\to \infty ,$$

where

(7) $$k^{*}\left(r\right)=k_{\infty }\left(1+de^{-\frac{r}{\gamma }}\right),$$

(8) $$\varepsilon \left(r\right)=\varepsilon_{\infty }\left(1+d^{*}e^{-\frac{r}{\gamma }}\right).$$

Model for hybrid nanofluid is [13]:

(9) $$\begin{array}{c} \mu_{hnf}=\frac{\mu_{f}}{{\left(1-\phi_{1}-\phi_{2}\right)}^{2.5}},\;\;\nu_{hnf}=\frac{\mu_{hnf}}{\rho_{hnf}},\;\;\rho_{hnf}=\rho_{f}\left(1-\phi_{1}-\phi_{2}\right)+\rho_{1}\phi_{1}+\rho_{2}\phi_{2},\\ \alpha_{hnf}=\frac{k_{hnf}}{{\left(\rho c_{p}\right)}_{hnf}},\;\;{\left(\rho c_{p}\right)}_{hnf}={\left(\rho c_{p}\right)}_{f}\left(1-\phi_{1}-\phi_{2}\right)+{\left(\rho c_{p}\right)}_{1}\phi_{1}+{\left(\rho c_{p}\right)}_{1}\phi_{1},\\ \frac{k_{hnf}}{k_{f}}=\frac{\phi_{1}k_{1}+\phi_{2}k_{2}+2\phi k_{f}+2\phi \left(\phi_{1}k_{1}+\phi_{2}k_{2}\right)-2{\left(\phi_{1}+\phi_{2}\right)}^{2}k_{f}}{\phi_{1}k_{1}+\phi_{2}k_{2}+2\phi k_{f}-\phi \left(\phi_{1}k_{1}+\phi_{2}k_{2}\right)+{\left(\phi_{1}+\phi_{2}\right)}^{2}k_{f}}. \end{array}$$

Here $\phi_{1}$ signifies solid volume fraction of $\mathrm{Si}O_{2}$, $\phi_{2}$ the solid volume fraction of ${\mathrm{MoS}}_{2}$, $\rho_{hnf}$ hybrid nanofluid density, ${\left(\rho c_{p}\right)}_{hnf}$ heat capacity of hybrid nanofluid, $\mu_{hnf}$ effective dynamic viscosity of hybrid nanofluid, $k_{hnf}$ the thermal conductivity of hybrid nanofluid, $\rho_{1}$ the density of $\mathrm{Si}O_{2}$, $\rho_{2}$ the density of ${\mathrm{MoS}}_{2}$, $k_{1}$ the thermal conductivity of $\mathrm{Si}O_{2}$, $k_{2}$ the thermal conductivity of ${\mathrm{MoS}}_{2}$, $k_{f}$ the thermal conductivity of base fluid, $\rho_{f}$ the density of base fluid, $k_{\infty }$ and $\varepsilon_{\infty }$ the constant permeability and porosity, d and $d^{*}$ the variable permeability and porosity, Q the heat generation/absorption, $C_{b}$ the drag coefficient, $\tilde{\sigma }$ the Stefan Boltzmann coefficient and k the mean absorption coefficient. Following Table 1 [14] consists of characteristics of base liquids and nanoparticles.

Considering

(10) $$\begin{array}{c} u=U_{w}=ae^{s/L}f^{\prime }(\zeta ),\;\;v=-\frac{R}{r+R}\sqrt{\frac{a\nu_{f}e^{s/L}}{2L}}\left(f(\zeta )+\zeta f^{\prime }(\zeta )\right),\;\;\zeta ={\left(\frac{ae^{s/L}}{2\nu_{f}L}\right)}^{1/2}r,\\ T=T_{\infty }+T_{0}e^{\frac{As}{2L}}\theta \left(\zeta \right),\;\;p=\rho_{f}a^{2}e^{2s/L}H\left(\zeta \right), \end{array}$$

we have

(11) $$\frac{1}{\left(1-\phi_{1}-\phi_{2}+\frac{\rho_{1}}{\rho_{f}}\phi_{1}+\frac{\rho_{2}}{\rho_{f}}\phi_{2}\right)}H^{{}^{\prime }}=\frac{1}{\zeta +K}f^{\prime^{2}},$$

(12) 1(1−ϕ1−ϕ2)2.5(1−ϕ1−ϕ2+ρ1ρfϕ1+ρ2ρfϕ2)(f‴+1ζ+Kf″−1(ζ+K)2f′−21σRes1+d*e−ζ1+de−ζf′)−ζ+2K(ζ+K)2K(f′)2+Kζ+Kff″+K(ζ+K)2ff′−2β(1+d*e−ζ)21+de−ζf′2=−1(1−ϕ1−ϕ2+ρ1ρfϕ1+ρ2ρfϕ2)Kζ+K(4H+ζH′)

(13) 1Pr1(1−ϕ1−ϕ2+(ρcp)1(ρcp)fϕ1+(ρcp)2(ρcp)fϕ2)khnfkf(θ″+1ζ+Kθ′)+Kζ+K(fθ′−Af′θ)+1(1−ϕ1−ϕ2+(ρcp)1(ρcp)fϕ1+(ρcp)2(ρcp)fϕ2)(2Q*θ+Ec(1−ϕ1−ϕ2)2.5((f″−1ζ+Kf′)2+2σRes1+d*e−ζ1+deζf′2)−43RdPr(((1+(θw−1)θ)3)θ′)′+2βEc(1−ϕ1−ϕ2+(ρcp)1(ρcp)fϕ1+(ρcp)2(ρcp)fϕ2)(1+d*e−ζ)21+de−ζf′3)=0,

(14) $$f=0,\;\;f^{\prime }=1,\;\;\theta =1\;\mathrm{at}\;\zeta =0,$$

(15) $$f^{\prime }\to 0,\;\;f^{\prime \prime }\to 0,\;\;\theta \to 0\;\mathrm{as}\;\zeta \to \infty .$$

Here Equation (1) is trivially verified. Eliminating pressure H from Equations (11) and (12), we have

(16) 1(1−ϕ1−ϕ2)2.5(1−ϕ1−ϕ2+ρ1ρfϕ1+ρ2ρfϕ2)(fiv+2ζ+Kf‴−1(ζ+K)2f″+1(ζ+K)3f′−21σRes1+d*e−ζ1+de−ζ(f″+1ζ+Kf′))+K(ζ+K)2ff″+Kζ+Kff‴−K(ζ+K)3ff′−3K(ζ+K)2f′2−3Kζ+Kf′f″−2β(1+d*e−ζ)21+de−ζ(2f′f″+1ζ+Kf′2)=0.

Here $Pe_{s}$ depicts the Peclet number, γ the parameter, $Re_{s}$ the local Reynolds number, β the inertia coefficient, Ec the Eckert number, K the curvature parameter, σ the permeability parameter, Rd the radiation parameter, $Q^{*}$ the heat generation/absorption parameter, Pr the Prandtl number, and Br the Brinkman number. These definitions are

(17) Pes=ResPr,  γ=αfLPes1/22νfL,  Res=uwLνf,  σ=k∞L2ε∞,  K=(aes/L2νfL)1/2R, β=Cbε∞2Lk∞,  Rd=4σT∞3k*kf,  θw=TwT∞,  Q*=QLuw(ρcp)f,  Ec=uw2(Tw−T∞) (cp)f, Pr=νfαf,  Br=PrEc.

## 3. Physical Quantities

Skin friction coefficient and local Nusselt number are given by

(18) (Res2)1/2Cf=1(1−ϕ1−ϕ2)2.5(f″(0)−1Kf′(0)),

(19) (Res2)−1/2Nus=−(khnfkf+43θw3Rd)θ′(0).

## 4. Entropy Generation

Entropy generation expression for considered flow problem is

(20) $$\begin{array}{cc} S_{gen}^{\prime \prime \prime }= & \underset{Thermal\;irreversibility}{\underset{︸}{\frac{k_{hnf}}{T_{m}^{2}}{\left(\frac{\partial T}{\partial r}\right)}^{2}}}+\underset{Viscous\;dissipation\;irreversibility}{\underset{︸}{\frac{\mu_{hnf}}{T_{m}}{\left(\frac{\partial u}{\partial r}-\frac{u}{r+R}\right)}^{2}}}+\underset{Heat\;generation/absorption\;irreversibility}{\underset{︸}{\frac{Q}{T_{m}}\left(T-T_{\infty }\right)}}+\\ \; & \underset{Thermal\;radiation\;irreversibility}{\underset{︸}{\frac{1}{T_{m}}\frac{\partial }{\partial r}\left(-\frac{16\sigma }{3k}T^{3}\frac{\partial T}{\partial r}\right)}}+\underset{Porous\;dissipation\;irreversibility}{\underset{︸}{\frac{\mu_{hnf}}{T_{m}}\frac{\varepsilon \left(r\right)}{k^{*}\left(r\right)}u^{2}+\frac{C_{b}\varepsilon^{2}\left(r\right)\;\rho_{hnf}}{T_{m}k^{{*}^{1/2}}}u^{3}}},\; \end{array}$$

Applying transformations (10) above expression reduces to

(21) Ng(ζ)=khnfkfα1θ′2+2PrQ*θ+43Rd((1+(θw−1)θ)3θ′)′+Br(1−ϕ1−ϕ2)2.5((f″−1ζ+Kf′)2+2σRes1+d*e−ζ1+deζf′2)+2βBr(1−ϕ1−ϕ2+ρ1ρfϕ1+ρ2ρfϕ2) (1+d*e−ζ)21+de−ζf′3,

in which $\alpha_{1}=\frac{\Delta T}{T_{m}}$ is the temperature difference parameter and $N_{g}=\frac{T_{m}}{\Delta T}S_{gen}^{\prime \prime \prime }\frac{2\nu_{f}L}{ae^{s/L}}$ the entropy generation rate.

## 5. Discussion

**Figure 2:**
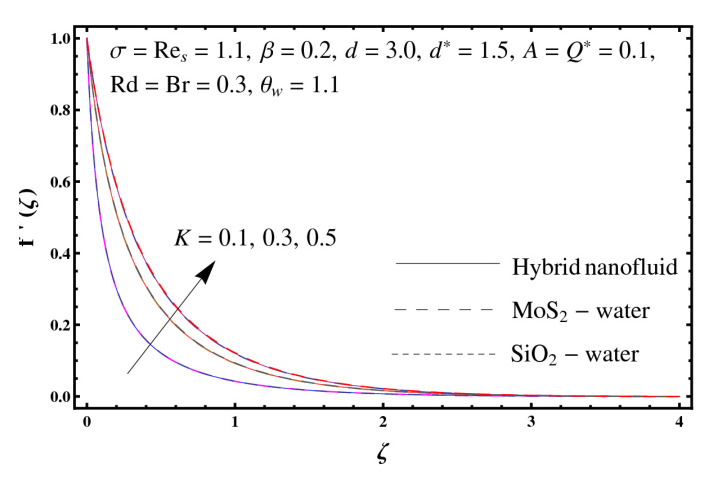
Sketch of $f^{\prime }\left(\zeta \right)$ against K.

**Figure 3:**
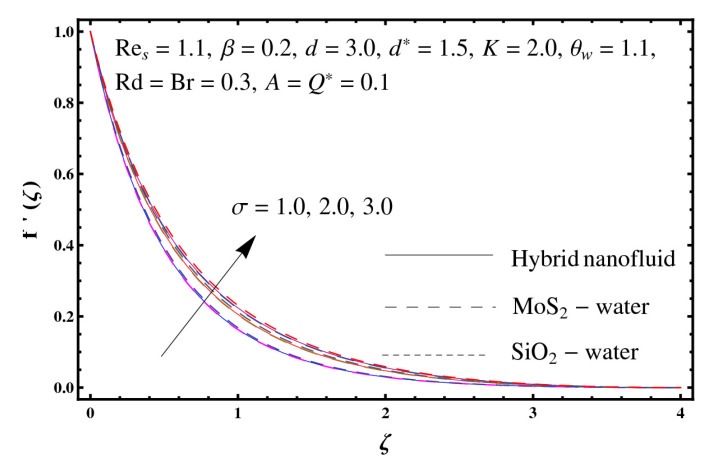
Sketch of $f^{\prime }\left(\zeta \right)$ against σ.

**Figure 4:**
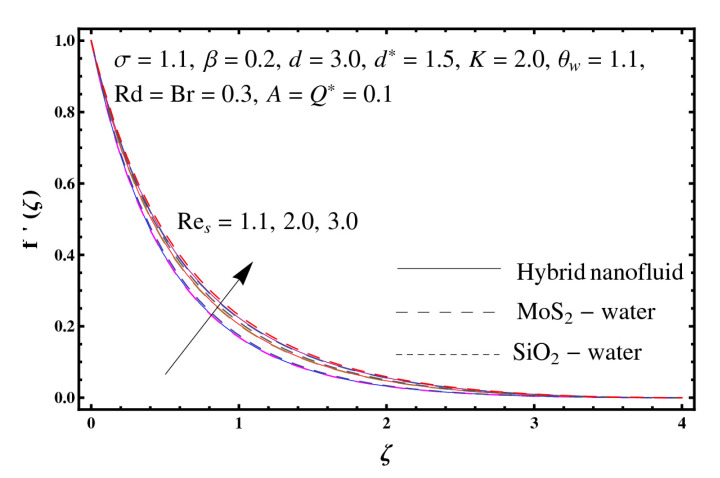
Sketch of $f^{\prime }\left(\zeta \right)$ against Res.

**Figure 5:**
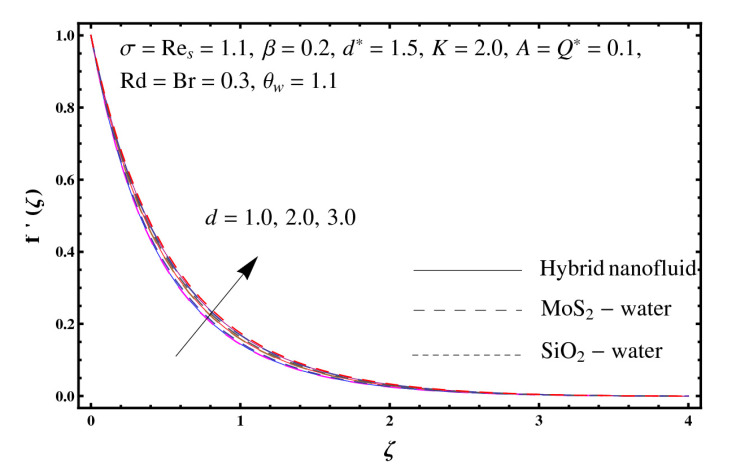
Sketch of $f^{\prime }\left(\zeta \right)$ against d.

**Figure 6:**
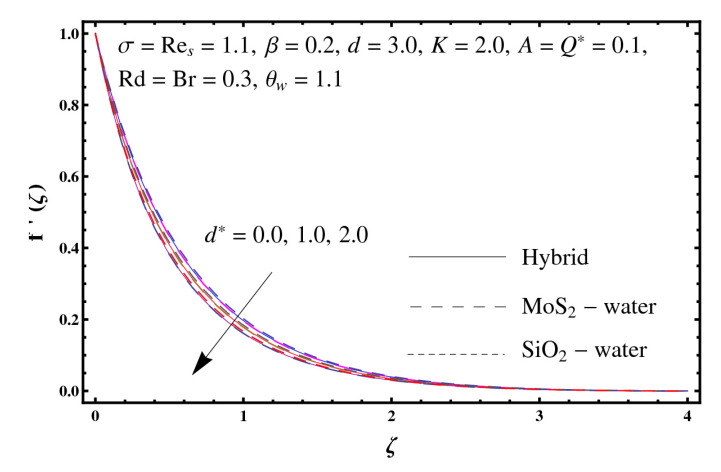
Sketch of $f^{\prime }\left(\zeta \right)$ against $d^{*}$.

**Figure 7:**
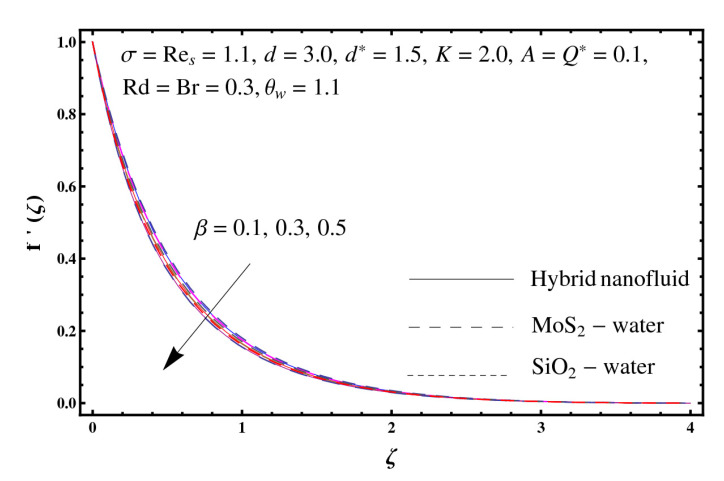
Sketch of $f^{\prime }\left(\zeta \right)$ against β.

**Figure 8:**
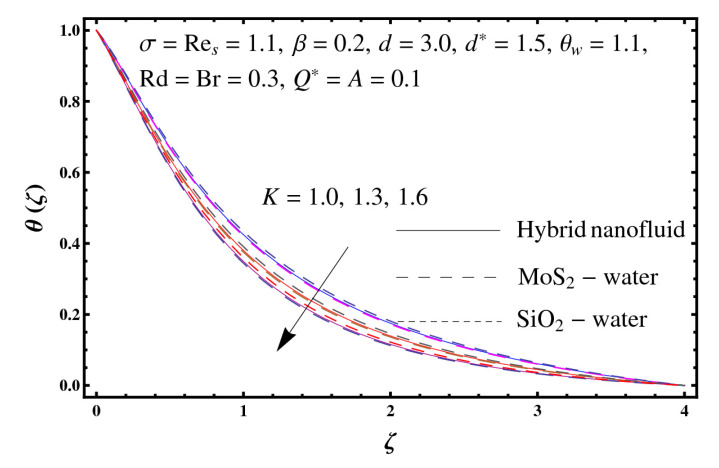
Sketch of θ(ζ) against K.

**Figure 9:**
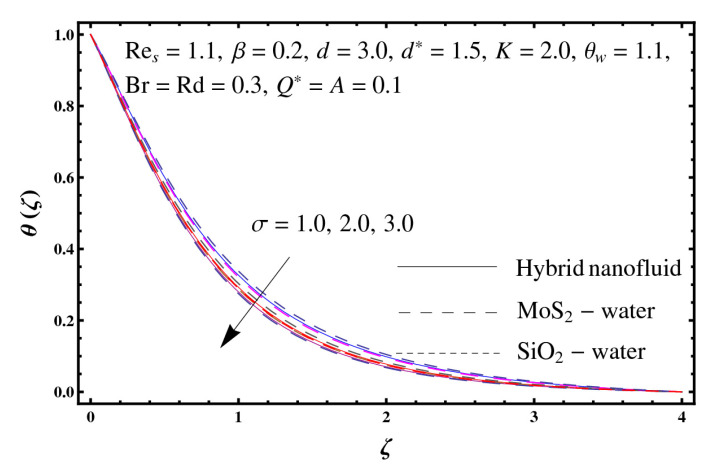
Sketch of θ(ζ) against σ.

**Figure 10:**
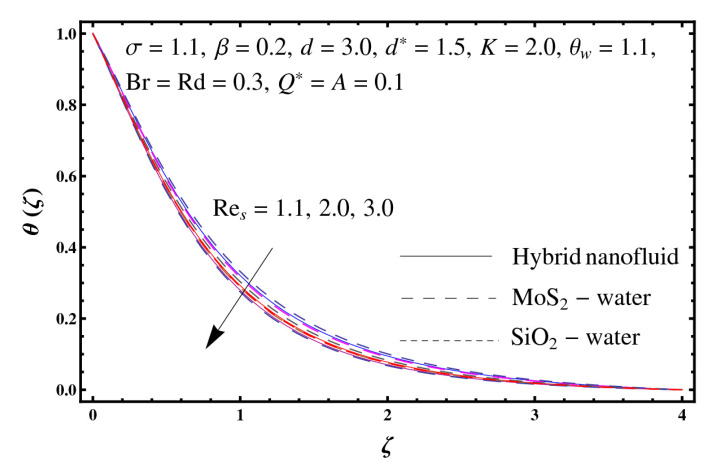
Sketch of θ(ζ) against Res.

**Figure 11:**
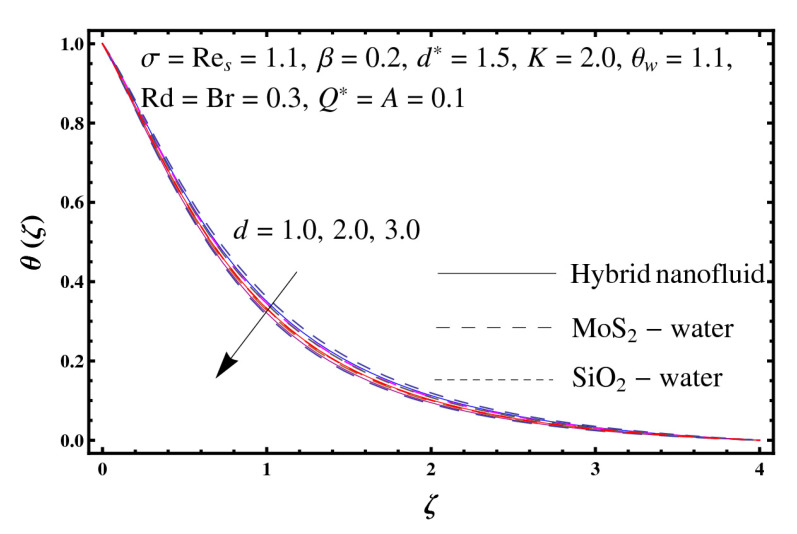
Sketch of θ(ζ) against d.

**Figure 12:**
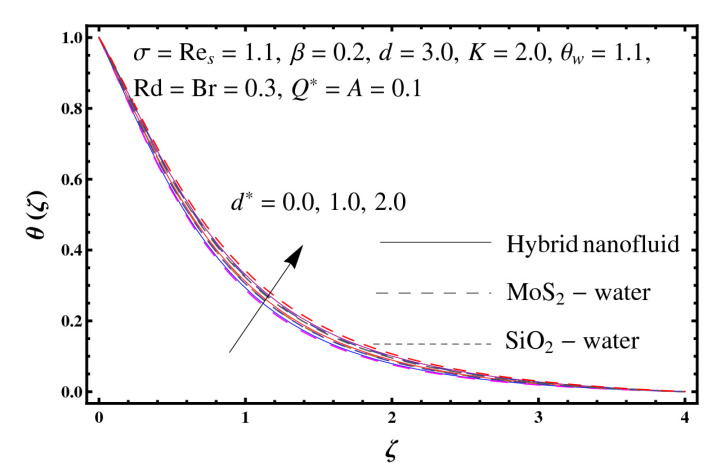
Sketch of θ(ζ) against $d^{*}$.

**Figure 13:**
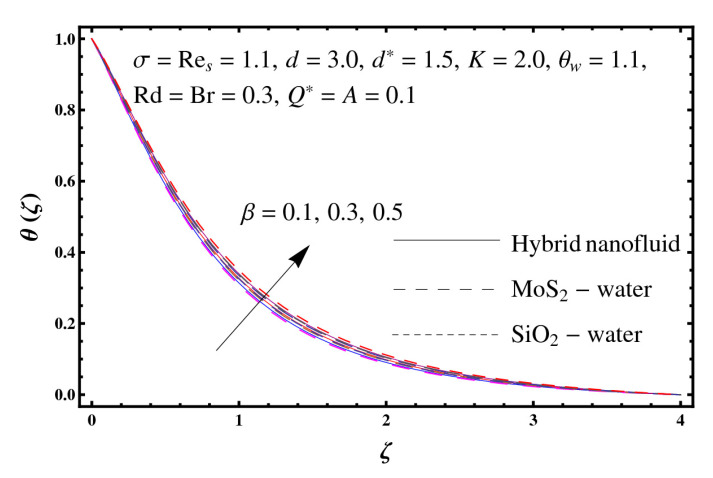
Sketch of θ(ζ) against β.

**Figure 14:**
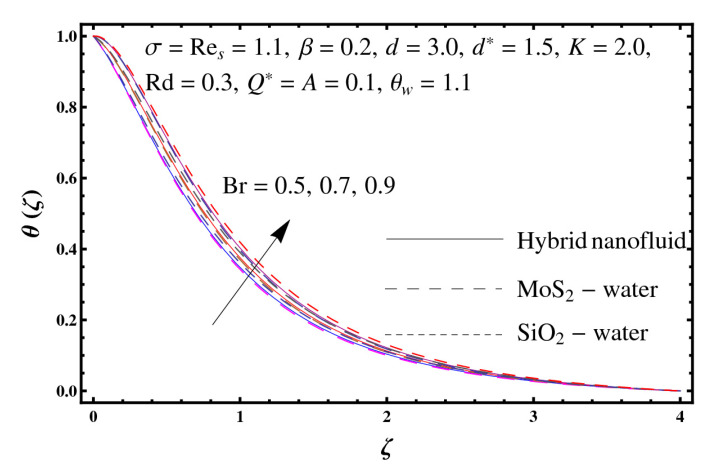
Sketch of θ(ζ) against Br.

**Figure 15:**
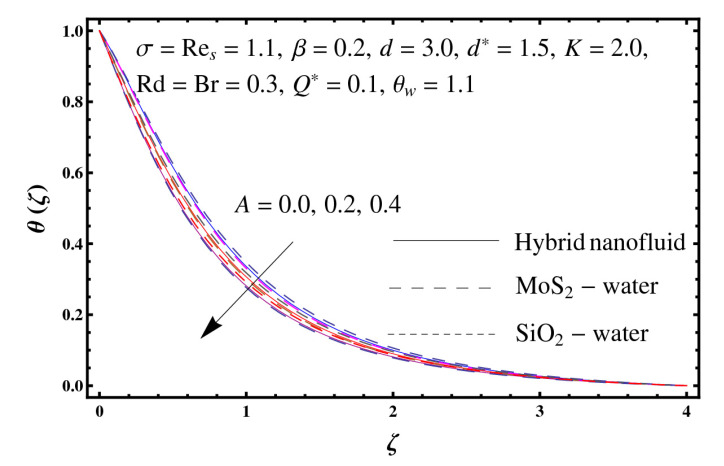
Sketch of θ(ζ) against A.

**Figure 16:**
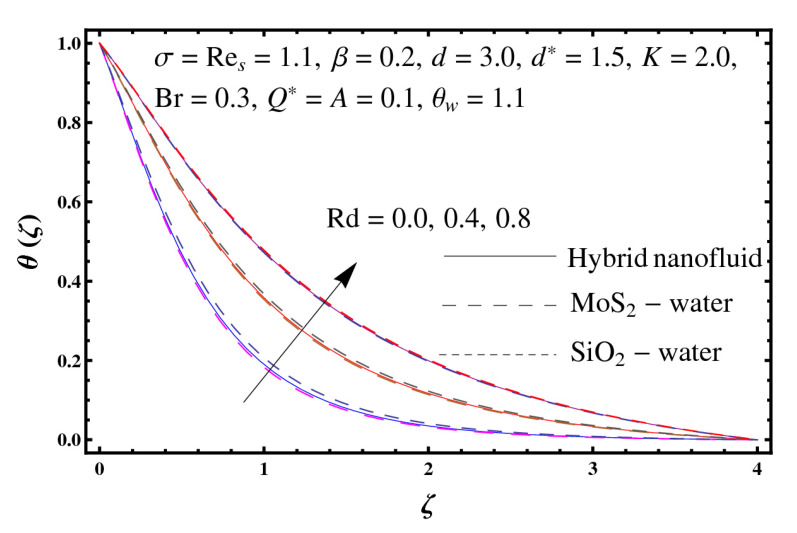
Sketch of θ(ζ) against Rd.

**Figure 17:**
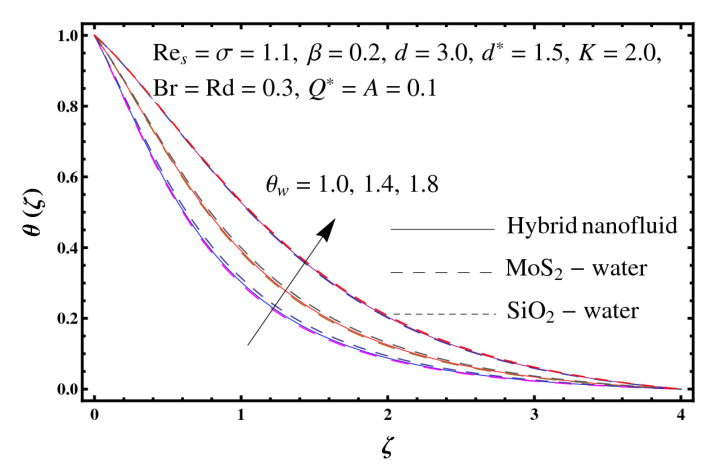
Sketch of θ(ζ) against $\theta_{w}$.

**Figure 18:**
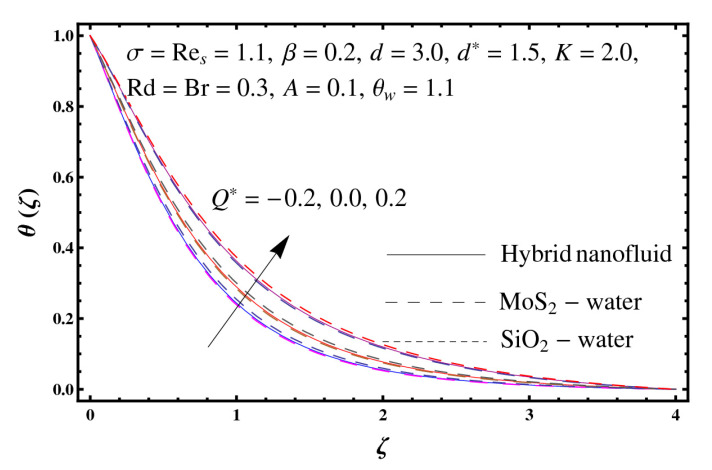
Sketch of θ(ζ) against $Q^{*}$.

**Figure 19:**
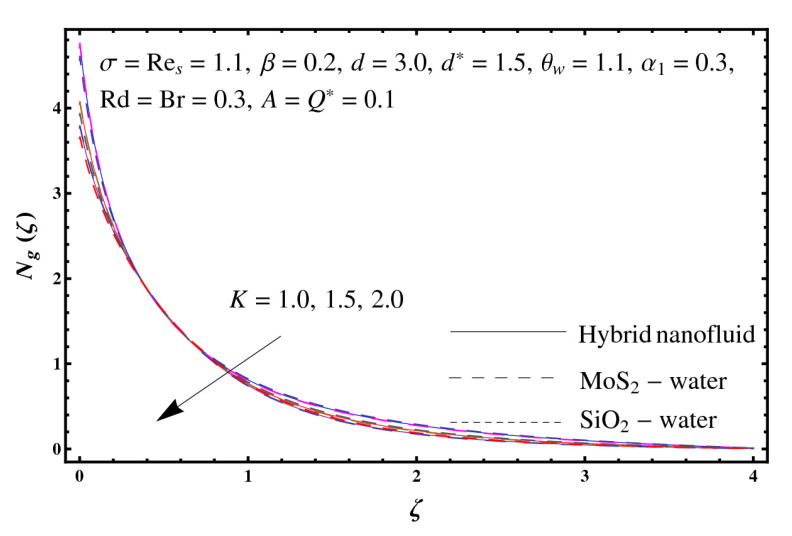
Sketch of $N_{g}\left(\zeta \right)$ against K.

**Figure 20:**
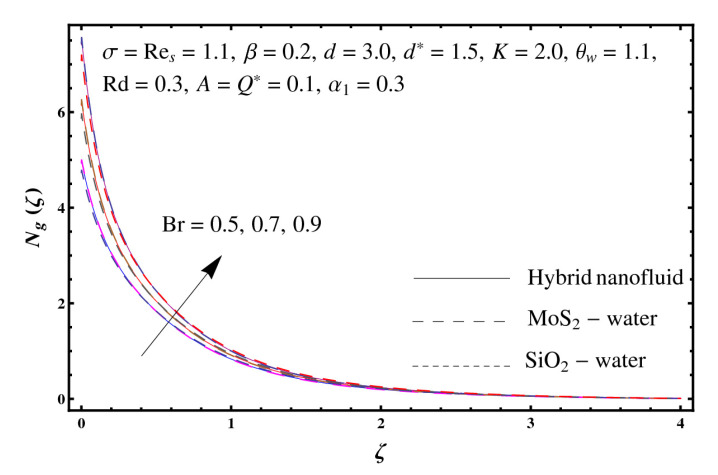
Sketch of $N_{g}\left(\zeta \right)$ against Br.

**Figure 21:**
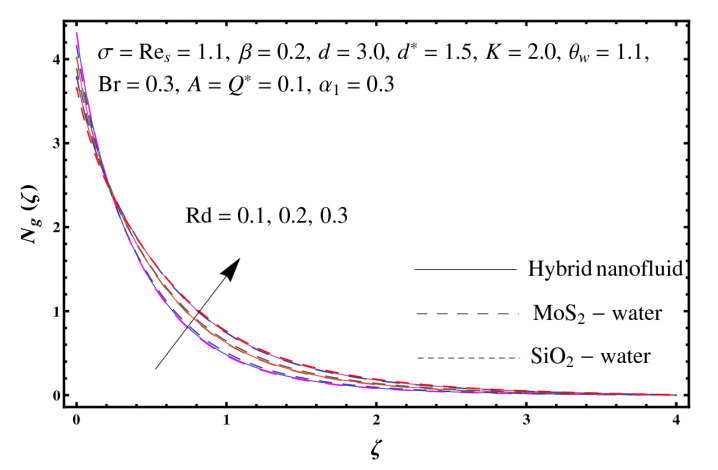
Sketch of $N_{g}\left(\zeta \right)$ against Rd.

**Figure 22:**
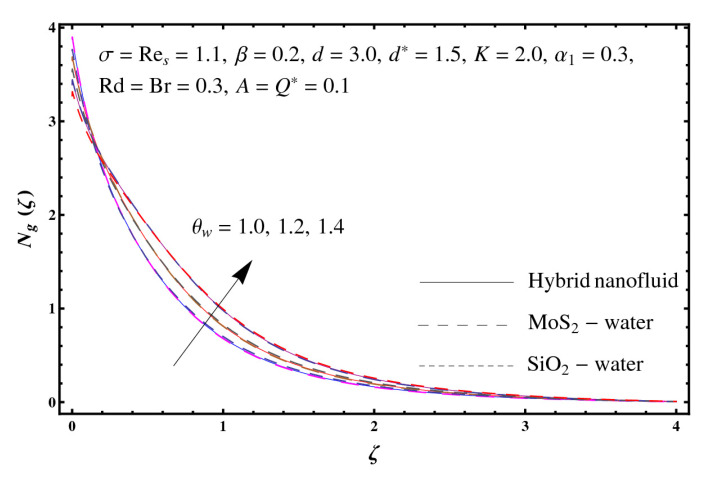
Sketch of $N_{g}\left(\zeta \right)$ against $\theta_{w}$.

**Figure 23:**
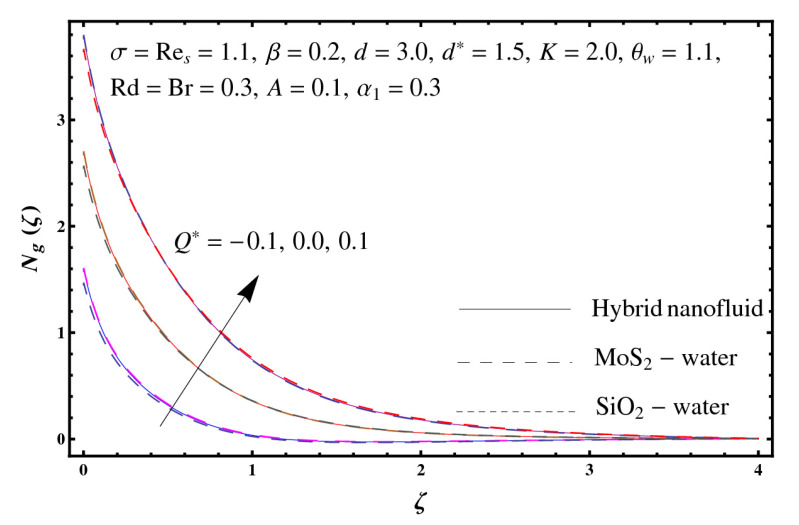
Sketch of $N_{g}\left(\zeta \right)$ against $Q^{*}$.

**Figure 24:**
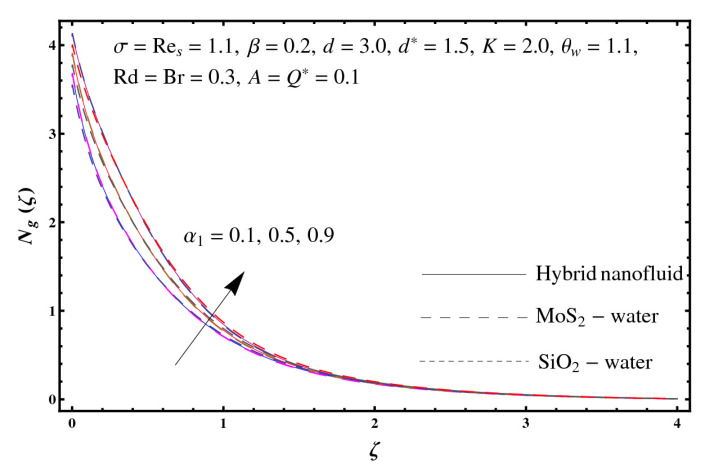
Sketch of $N_{g}\left(\zeta \right)$ against $\alpha_{1}$.

This section interprets the characteristics of velocity $f^{\prime }\left(\zeta \right),$ temperature θ(ζ) and entropy generation rate $N_{g}\left(\zeta \right)$ through curvature parameter (K), porosity parameter (σ), Reynolds number $\left(Re_{s}\right),$ variable porosity and permeability parameters (d) and $\left(d^{*}\right),$ inertia coefficient (β), Brinkman number (Br), temperature exponent (A), temperature ratio parameter $\left(\theta_{w}\right)$, radiation parameter (Rd), and heat generation/absorption parameter $\left(Q^{*}\right).$ Comparative results are obtained for hybrid nanofluid (MoS_2_-SiO_2_/water) and nanofluid (MoS_2_/water and SiO_2_/ water). The consequences of $f^{\prime }\left(\zeta \right)$ against (K) are in Figure 2. An enhancement in $f^{\prime }\left(\zeta \right)$ is observed through (K) for both hybrid nanofluid and nanomaterial. Physically the bend of the curved stretching sheet contributes in accelerating the flow. The impact of (σ) on $f^{\prime }\left(\zeta \right)$ is illustrated in Figure 3. Here $f^{\prime }\left(\zeta \right)$ is an increasing function of (σ) for both hybrid nanofluid and nanofluid. Velocity $f^{\prime }\left(\zeta \right)$ through $\left(Re_{s}\right)$ is drawn in Figure 4. Higher $\left(Re_{s}\right)$ correspond to stronger $f^{\prime }\left(\zeta \right)$ for both hybrid nanofluid and nanofluid. Physically (Res) has a direct relation with inertial forces due to which the velocity increases. Reverse trend of $f^{\prime }\left(\zeta \right)$ is noted for (d) and $\left(d^{*}\right)$ in both hybrid nanofluid and nanofluid (see Figure 5 and Figure 6). Figure 7 is plotted for the features of $f^{\prime }\left(\zeta \right)$ through (β). Higher estimation of (β) lead to a reduction in $f^{\prime }\left(\zeta \right)$ for both hybrid nanofluid (MoS_2_-SiO_2_/water) and nanofluid (MoS_2_/water and SiO_2_/water). Figure 8 addressed θ(ζ) against (K). By increasing (K) reduction is observed through (K) for both hybrid nanofluid and nanofluid. Figure 9 captured consequences of θ(ζ) against (σ). Here reduction in θ(ζ) is analyzed through higher (σ) for both hybrid nanofluid and nanofluid. Figure 10 depicts that θ(ζ) is a decreasing function of $\left(Re_{s}\right)$ for both hybrid nanofluid (MoS_2_-SiO_2_/water) and nanofluid (MoS_2_/water and SiO_2_/ water). Behaviors of θ(ζ) through (d) and $\left(d^{*}\right)$ is portrayed in Figure 11 and Figure 12. An enhancement in θ(ζ) is observed through $\left(d^{*}\right)$ while opposite trend is seen against (d) for both hybrid nanofluid and nanofluid. Aspects of θ(ζ) against (β) is deliberated in Figure 13. Higher (β) produces resilience in the fluid motion due to which more heat is produced which strengthens the thermal field θ(ζ) for both hybrid nanofluid (MoS_2_-SiO_2_/water) and nanofluid (MoS_2_/water and SiO_2_/ water). Figure 14 cleared that θ(ζ) is an increasing function of (Br) for both hybrid nanofluid and nanofluid. Physically (Br) has a direct relation with heat generation by fluid friction which causes stronger θ(ζ). Significant behavior of θ(ζ) through (A) is drawn in Figure 15. Higher (A) produces weaker θ(ζ) in both hybrid nanofluid and nanofluid. Curves of θ(ζ) against (Rd) is elucidated in Figure 16. Higher estimation of (Rd) strengthen θ(ζ) and more related layer thickness for both hybrid nanofluid and nanofluid. Variation of θ(ζ) through $\left(\theta_{w}\right)$ is displayed in Figure 17. It is seen that higher $\left(\theta_{w}\right)$ enhance θ(ζ) for both hybrid nanofluid (MoS_2_-SiO_2_/water) and nanofluid (MoS_2_/water and SiO_2_/ water). Role of $\left(Q^{*}\right)$ on θ(ζ) is highlighted in Figure 18. Here an augmentation in θ(ζ) is observed through $\left(Q^{*}\right)$ for both hybrid nanofluid and nanofluid. Influence of (K) on $N_{g}\left(\zeta \right)$ is depicted in Figure 19. Entropy generation rate decreases due to higher (K) for both hybrid nanofluid and nanofluid. Figure 20 and Figure 21 analyzed the behavior of $N_{g}\left(\zeta \right)$ against (Br) and (Rd). Similar trend of $N_{g}\left(\zeta \right)$ is witnessed through (Br) and (Rd) for both hybrid nanofluid and nanomaterial. Figure 22 illustrates that $N_{g}\left(\zeta \right)$ increases for higher $\left(\theta_{w}\right)$ for both hybrid nanofluid (MoS_2_-SiO_2_/water) and nanofluid (MoS_2_/water and SiO_2_/water). Impact of $\left(Q^{*}\right)$ on $N_{g}\left(\zeta \right)$ is sketched in Figure 23. Higher $\left(Q^{*}\right)$ produces augmentation in θ(ζ) due to rise in surface temperature for both hybrid nanofluid and nanofluid. Consequences of $\left(\alpha_{1}\right)$ on $N_{g}\left(\zeta \right)$ is highlighted in Figure 24. Here $N_{g}\left(\zeta \right)$ is an increasing function of $\left(\alpha_{1}\right)$ for both hybrid nanofluid and nanofluid. Contribution of involved variables on skin friction coefficient ${\left(\frac{Re_{s}}{2}\right)}^{1/2}C_{f}$ is displayed in Table 2 Reduction in ${\left(\frac{Re_{s}}{2}\right)}^{1/2}C_{f}$ is seen through (K), (σ), $\left(Re_{s}\right),$ (d) and (β) for both hybrid nanofluid and nanofluid. Significant behavior of ${\left(\frac{Re_{s}}{2}\right)}^{-1/2}Nu_{s}$ through influential variables is shown in Table 3 Here (K), (σ), (d), $\left(R_{d}\right)$ and $\left(\theta_{w}\right)$ strengthen the ${\left(\frac{Re_{s}}{2}\right)}^{-1/2}Nu_{s}$ for both hybrid nanofluid and nanofluid. Table 4 is drawn to compare the values of skin friction coefficient with Okechi et al. [28]. It is analyzed that present results are in good agreement with those presented in ref. [28].

## 6. Conclusions

The main points of the current analysis are:

- Velocity has the opposite scenario for variable characteristics of porosity and permeability.
- Aspects of the permeability parameter on velocity are reversed when compared with the thermal field.
- Enhancement in velocity is witnessed against the curvature parameter.
- Temperature against Brinkman number and radiation parameter have a similar trend.
- Augmentation in the thermal field is observed through the inertia coefficient.
- Entropy generation rate increases for heat generation/absorption and temperature ratio parameter.
- Skin friction coefficient for variable permeability parameter decays.
- Augmentation in local Nusselt number is witnessed for radiation and temperature ratio parameters.
- Some possible extension of the current analysis may be as follows:
- Importance of melting heat transfer effects inflow of hybrid nanofluid.
- Binary chemical reaction and activation energy aspects inflow by curved stretching surface.
- Modeling of non-Newtonian liquids inflow due to curved geometry.

## Figures and Tables

**Table 1:** Physical properties of base and nanoparticles.

Physical Properties	Base Fluid	Nanoparticles	
	H_2_O	SiO_2_	MoS_2_
ρ (kg/m^3^)	997.1	2650	5060
k (W/mK)	0.613	1.5	34.5
C_p_ (J/kgK)	4179	730	397.746

**Table 2:** Numerical data of skin friction coefficient ${\left(\frac{Re_{s}}{2}\right)}^{1/2}C_{f}$ for K, σ, $Re_{s},$ d, $d^{*}$, and β.

K	σ	Res	d	$d^{*}$	β	(Res2)1/2Cf		
						Hybrid Nanofluid	MoS_2_-Water	SiO_2_-Water
1.0	1.1	1.1	3.0	1.5	0.2	3.84238	3.85225	3.79266
1.3						3.41971	3.43047	3.36545
1.5						3.23852	3.24971	3.18205
2.0	1.0	1.1	3.0	1.5	0.2	2.99600	3.00776	2.93661
	2.0					2.75157	2.76442	2.68650
	3.0					2.65921	2.67253	2.59173
2.0	1.1	1.1	3.0	1.5	0.2	2.95453	2.96646	2.89425
		1.5				2.83785	2.85030	2.77490
		1.9				2.76557	2.77835	2.70085
2.0	1.1	1.1	1.0	1.5	0.2	3.24720	3.25959	3.18459
			2.0			3.06787	3.07997	3.00672
			3.0			2.95453	2.96646	2.89425
2.0	1.1	1.1	3.0	0.0	0.2	2.64363	2.65338	2.59446
				1.0		2.84189	2.85292	2.78613
				2.0		3.07546	3.08842	3.00990
2.0	1.1	1.1	3.0	1.5	0.0	2.74260	2.75107	2.69994
					0.1	2.85047	2.86073	2.79873
					0.3	3.05515	3.06865	2.98681

**Table 3:** Numerical data of local Nusselt number ${\left(\frac{Re_{s}}{2}\right)}^{-1/2}Nu$ for K, σ, $Re_{s},$ d, $d^{*}$, β, Br, Rd, $\theta_{w}$ and $Q^{*}$.

K	σ	$Re_{s}$	d	$d^{*}$	β	Br	Rd	$\theta_{w}$	$Q^{*}$	(Res2)−1/2Nu		
										Hybrid Nanofluid	MoS_2_-Water	SiO_2_-Water
1.0	1.1	1.1	3.0	1.5	0.2	0.3	0.3	1.1	0.1	0.78812	0.77888	0.78773
1.3										0.95609	0.94689	0.96164
1.6										1.06312	1.05373	1.07280
2.0	1.0	1.1	3.0	1.5	0.2	0.3	0.3	1.1	0.1	1.12722	1.11800	1.13864
	2.0									1.29904	1.28665	1.32381
	3.0									1.36435	1.35064	1.39455
2.0	1.1	1.1	3.0	1.5	0.2	0.3	0.3	1.1	0.1	1.15619	1.14646	1.16977
		2.0								1.29904	1.28665	1.32381
		3.0								1.36435	1.35064	1.39455
2.0	1.1	1.1	3.0	0.0	0.2	0.3	0.3	1.1	0.1	1.36447	1.35379	1.38047
				1.0						1.23024	1.22030	1.24414
				2.0						1.07829	1.06870	1.09196
2.0	1.1	1.1	3.0	1.5	0.0	0.3	0.3	1.1	0.1	1.28077	1.27229	1.28646
					0.1					1.21696	1.20783	1.22679
					0.3					1.09811	1.08785	1.11512
2.0	1.1	1.1	3.0	1.5	0.2	0.5	0.3	1.1	0.1	0.75746	0.75223	0.74922
						0.6				0.55799	0.55499	0.53882
						0.7				0.35843	0.35767	0.32834
2.0	1.1	1.1	3.0	1.5	0.2	0.3	0.0	1.1	0.1	1.09425	1.08172	1.11263
							0.4			1.16916	1.15964	1.18257
							0.8			1.22079	1.21096	1.23532
2.0	1.1	1.1	3.0	1.5	0.2	0.3	0.3	1.0	0.1	1.14395	1.13379	1.15834
								1.4		1.18662	1.17787	1.19835
								1.8		1.19871	1.19025	1.21112
2.0	1.1	1.1	3.0	1.5	0.2	0.3	0.3	1.1	−0.2	1.55119	1.53599	1.57635
									0.0	1.30480	1.29301	1.32259
									0.2	0.97895	0.97161	0.98815

**Table 4:** Comparative values of skin friction coefficient ${\left(\frac{Re_{s}}{2}\right)}^{1/2}C_{f}$ for distinct values of K.

K	−(Res2)1/2Cf	
	Okechi et al. [28]	Present
5	1.4196	1.45703
10	1.3467	1.36819
20	1.3135	1.32810
30	1.3028	1.31536
40	1.2975	1.30912
50	1.2944	1.30539
100	1.2881	1.29804
200	1.2850	1.29443
1000	1.2826	1.29152

## Data Availability

Data is contained within the article.

## Nomenclature

R	Radius of curvature	s,r	space coordinate
u,v	velocity components	$U_{w}$	surface stretching velocity
$\rho_{1},\rho_{2}$	densities of nanaparticles	$\mu_{f}$	fluid dynamic viscosity
*d*	variable permeability	$k_{1},k_{2}$	thermal conductivity of nanoparticle
$\rho_{f}$	fluid density	$d^{*}$	variable porosity
$\nu_{f}$	kinematic fluid viscosity	$\nu_{hnf}$	kinematic viscosity of hybrid nanofluid
$k_{f}$	basefluid thermal conductivity	$k_{hnf}$	thermal conductivity of hybrid nanofluid
$\alpha_{f}$	thermal diffusivity of base fluid	$T_{w}$	surface temperature
$\alpha_{hnf}$	thermal diffusivity of hybrid nanofluid	$T_{\infty }$	ambient temperature
ε	porosity	$k^{*}$	permeability of porous medium
p	pressure	$C_{b}$	drag coefficient
Q	heat generation/absorption	$\tilde{\sigma }$	Stefan Boltzmann constant
k	mean absorption coefficient	$Q^{*}$	heat generation/absorption parameter
F	non-uniform inertia coefficient	$\phi_{1},\phi_{2}$	solid volume fraction of nanoparticles
Rd	radiation parameter	Res	local Reynolds number
β	Inertia coefficient	Br	Brinkman number
$C_{f}$	skin friction coefficient	$Nu_{s}$	local Nusselt number
σ	local porosity parameter	$Pe_{s}$	Peclet number
θ	dimensionless temperature	$f^{\prime }$	dimensionless velocity
K	curvature parameter	$N_{g}\left(\zeta \right)$	entropy generation rate
H	dimensionless pressure	ζ	dimensionless variable
Pr	Prandtl number	A	temperature exponent
